# Association between number of medications and hip fractures in Japanese elderly using conditional logistic LASSO regression

**DOI:** 10.1038/s41598-023-43876-3

**Published:** 2023-10-06

**Authors:** Takuya Uematsu, Yuta Kawakami, Shuko Nojiri, Tomoyuki Saito, Yoshiki Irie, Takatoshi Kasai, Yoshimune Hiratsuka, Muneaki Ishijima, Manabu Kuroki, Hiroyuki Daida, Yuji Nishizaki

**Affiliations:** 1 Clinical Translational Science, Juntendo University School of Medicine Graduate School of Medicine, 2-1-1 Hongo, Bunkyo-ku, Tokyo, 113-8421 Japan; 2 Department of Hospital Pharmacy, Juntendo University Hospital, Tokyo, Japan; 3 Medical Technology Innovation Center, Juntendo University, Tokyo, Japan; 4 Clinical Research and Trial Center, Juntendo University, Tokyo, Japan; 5 Graduate School of Engineering Science, Tokyo University of Science, Tokyo, Japan; 6 Department of Cardiology, Juntendo University School of Medicine Graduate School of Medicine, Tokyo, Japan; 7 Department of Ophthalmology, Juntendo University School of Medicine Graduate School of Medicine, Tokyo, Japan; 8 Department of Medicine for Orthopedics and Motor Organ, Juntendo University School of Medicine Graduate School of Medicine, Tokyo, Japan; 9 Graduate School of Engineering Science, Yokohama National University, Kanagawa, Japan

**Keywords:** Diseases, Risk factors

## Abstract

To examine the association between hip fracture and associated factors, including polypharmacy, and develop an optimal predictive model, we conducted a population-based matched case–control study using the health insurance claims data on hip fracture among Japanese patients. We included 34,717 hospitalized Japanese patients aged ≥ 65 years with hip fracture and 34,717 age- and sex- matched controls who were matched 1:1. This study included 69,434 participants. Overall, 16 variable comorbidities and 60 variable concomitant medications were used as explanatory variables. The participants were added to early elderly and late elderly categories for further analysis. The odds ratio of hip fracture increased with the number of medications only in the early elderly. AUC was highest for early elderly (AUC, 0.74, 95% CI 0.72–0.76). Use of anti-Parkinson’s drugs had the largest coefficient and was the most influential variable in many categories. This study confirmed the association between risk factors, including polypharmacy and hip fracture. The risk of hip fracture increased with an increase in medication number taken by the early elderly and showed good predictive accuracy, whereas there was no such association in the late elderly. Therefore, the early elderly in Japan should be an active target population for hip fracture prevention.

## Introduction

The world’s population is aging rapidly, and Japan may become a super-aging society unlike other countries. Due to this trend, the national health care costs are continually increasing, which reached 44.4 trillion yen in 2019. Further, elderly individuals aged ≥ 65 years are responsible for 61% of the total cost^1^. Indeed, with the aging of elderly individuals and the development of several chronic diseases, including lifestyle-related disorders, the number of visits to multiple hospitals and departments increases. This trend results in polypharmacy, which is defined as the simultaneous prescription of multiple drugs.

Polypharmacy is associated with several issues. Of which, the most serious ones are adverse medication event induced by drug interactions, poor adherence, and duplicate administration of the same prescribed drugs^2,3^. If elderly individuals take a higher number of medications, such as muscle relaxant benzodiazepines and cardiovascular drugs, which cause dizziness and lightheadedness, the risk of fall increases^4,5^. Inappropriate medications that may have risks that outweigh benefits are referred to as potentially inappropriate medications (PIMs)^6–8^, The use of PIMs can lead to adverse events such as falls, fractures, and delirium in elderly individuals. Hence, it is important to reduce or eliminate their application. Furthermore, even with a slight external force, elderly people are more likely to develop fragility fractures caused by osteoporosis or other similar conditions. Hip fracture is frequently associated with decreased walking ability.

Hip fracture is a high-risk fracture that often occurs in elderly patients with osteoporosis^9–11^, thereby resulting in decreased performance of activities of daily living and eventual state of being bedridden or the need for long-term care. This causes not only low quality of life but also significant health economic concerns, such as high societal expenditures, since the national health care costs continually increased in recent years. The number of new hip fracture cases in Japan was 190,000 in 2017, with an estimated number of 320,000 at peak in 2042^12^ and 240,000 in 2050^11,13^.

Therefore, several studies using methods such as logistic regression have been used to assess the risk factor and prognosis of hip fracture^14–17^. By contrast, classical generalized linear models including logistic regression are used to estimate a model by fitting a straight line to the data. Although they are excellent predictive models, they have disadvantages such as multicollinearity and overfitting in the presence of several explanatory variables, such as dimensional data^18^. To suppress overfitting, Tibshirani proposed the use of the least absolute shrinkage and selection operator (LASSO), which inserts a regularization term in loss function to improve generalization performance, prediction accuracy, and interpretability. LASSO can simplify the model by forcing less influential variables to have zero influence among many explanatory variables^18–20^. Recently, machine learning-based predictive models have been used frequently in the field of medicine worldwide^21–24^. Therefore, we believe that LASSO is suitable for analyzing the data with a high number of parameters, such as the data collected in this study, as it enables variable selection and simplifies the model. However, there are only few studies using machine learning in the medical field in Japan. In the assessment of risk factors, including polypharmacy for hip fracture, classical statistical approaches are utilized for small-scale data with minimal generalizability, and there are no reports using machine learning methods for large-scale data. It can efficiently select predictors for regression models with several covariates, such as concomitant medications and comorbidities, and can construct simple and optimal predictive models. This can provide clinicians with a model that is simple to use in clinical settings and easy to interpret for everyone.

This study created a prediction model for hip fracture using conditional LASSO and investigated the association between hip fracture and risk factors, including polypharmacy in Japan, a hyper-aged society. We used conditional LASSO because of its matched case–control study design, ability to optimize variable selection, and regularization for high-dimensional data with many variables, such as diagnosis procedure combination (DPC) in administrative database, and ability to pursue high prediction accuracy and interpretability.

## Result

### Characteristics of the participants

**Figure 1:**
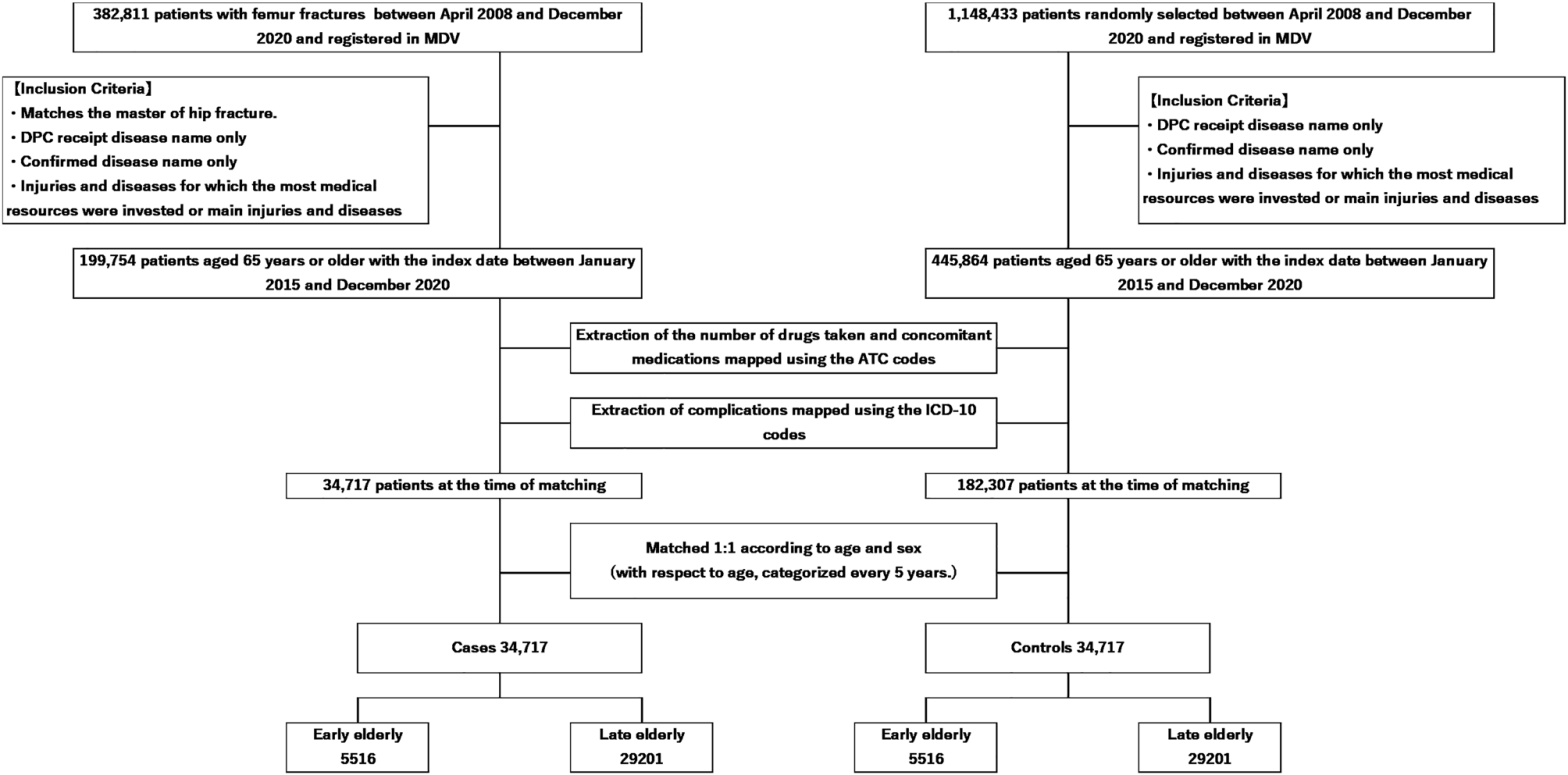
Flowchart showing the recruitment procedure for cases and controls. MDV, Medical Data Vision.

Based on MDV data from approximately 1 million people, 199,754 cases and 445,864 controls were selected, fulfilling the criteria for “Selection of Cases and Controls.” Then, 34,717 cases and 182,307 controls meeting the criteria for “Definition of concomitant medications and comorbidities” were extracted. Finally, a 1:1 matching based on age and sex was performed, resulting in the inclusion of 69,434 participants, with 34,717 cases and 34,717 controls (Fig. 1). If there were multiple controls for a case, they were randomly selected. Among them, 15.9% were early elderly and 84.1% were late elderly. Approximately 71.4% of patients were women, and 28.6% were men. The highest and second highest number of medications taken was 1 or 2 in the case and control groups, respectively. The mean numbers of medications were 5.8 ± 4.2 in the case group and 5.3 ± 4.0 in the control group. The mean numbers of comorbidities in the case and control groups were of 7.4 ± 3.6 and 6.4 ± 3.3, respectively. The comorbidities and concomitant medications of the participants are shown in Table 1. The characteristics of the early elderly and late elderly are shown in Appendix 3.

**Table 1:** Characteristics of patients with hip fracture and controls SD, standard deviation; Std diff, standardized difference. (a) Number of medications and Others. (b) Comorbidities and Concomitant medications.

(A)			
	Case group	Control group	Std diff
	(n = 34,717)	(n = 34,717)	
	N (%)	N (%)	
Age category Early elderly	5516 (15.9)	5516 (15.9)	–
Late elderly	29,201 (84.1)	29,201 (84.1)	–
Age Mean (SD)	82.7 (7.6)	82.6 (7.6)	–
Sex Male	9920 (28.6)	9920 (28.6)	–
Female	24,797 (71.4)	24,797 (71.4)	–
Number of medications			
0	935 (2.7)	2149 (6.2)	−0.17
1	4410 (12.7)	4699 (13.5)	−0.024
2	3876 (11.2)	4022 (11.6)	−0.013
3	3342 (9.6)	3296 (9.5)	0.003
4	3132 (9.0)	3183 (9.2)	−0.007
5	2889 (8.3)	2998 (8.6)	−0.011
6	2780 (8.0)	2737 (7.9)	0.004
7	2628 (7.6)	2466 (7.1)	0.019
8	2338 (6.7)	2079 (6.0)	0.029
9	2021 (5.8)	1734 (5.0)	0.035
10	1659 (4.8)	1465 (4.2)	0.029
11	1260 (3.6)	1126 (3.2)	0.022
12	982 (2.8)	828 (2.4)	0.025
13	698 (2.0)	592 (1.7)	0.022
14	558 (1.6)	421 (1.2)	0.034
≥ 15	1209 (3.5)	922 (2.7)	0.046
Number of medications			
Mean (SD)	5.8 (4.2)	5.3 (4.0)	0.13
Number of comorbidities			
Mean (SD)	7.4 (3.6)	6.4 (3.3)	0.27

(B)			
	Case group	Control group	Std diff
	(n = 34,717)	(n = 34,717)	
	N (%)	N (%)	
Comorbidities			
Diseases of the circulatory system	27,308 (78.7)	27,447 (79.1)	−0.01
Digestive system diseases	25,615 (73.8)	22,895 (65.9)	0.171
Endocrine, nutritional, and metabolic diseases	22,754 (65.5)	21,644 (62.3)	0.067
Respiratory system diseases	20,180 (58.1)	18,402 (53.0)	0.103
Neoplasms	19,446 (56.0)	18,394 (53.0)	0.061
Symptoms and signs and abnormal clinical and laboratory findings, not elsewhere classified	17,640 (50.8)	16,167 (46.4)	0.085
Certain infectious and parasitic diseases	16,504 (47.5)	14,279 (41.1)	0.129
Genitourinary system diseases	16,485 (47.5)	14,732 (42.4)	0.102
Nervous system diseases	15,848 (45.6)	12,562 (36.2)	0.193
Diseases of the blood and blood-forming organs and certain disorders involving the immune mechanism	14,597 (42.0)	13,516 (38.9)	0.063
Concomitant medications			
Antacids, antiflatulents, and anti-ulcers	20,275 (58.4)	21,326 (61.4)	−0.062
Antithrombotic agents	11,626 (33.5)	11,088 (31.9)	0.033
Calcium antagonists	10,803 (31.1)	10,103 (29.1)	0.044
Agents acting on the renin-angiotensin system	9659 (27.8)	8996 (25.9)	0.043
Psycholeptics	8468 (24.4)	9273 (26.7)	−0.053
Lipid-regulating/anti-atheroma preparations	7726 (22.3)	7406 (21.3)	0.022
Diuretics	7579 (21.8)	7478 (21.5)	0.007
Vitamins	6510 (18.7)	5296 (15.3)	0.093
Drugs for constipation	5724 (16.5)	8040 (23.2)	−0.168
Antidiabetic drugs	5322 (15.3)	4057 (11.7)	0.107

### Association between the number of medications and risk of hip fracture stratified according to age

**Figure 2:**
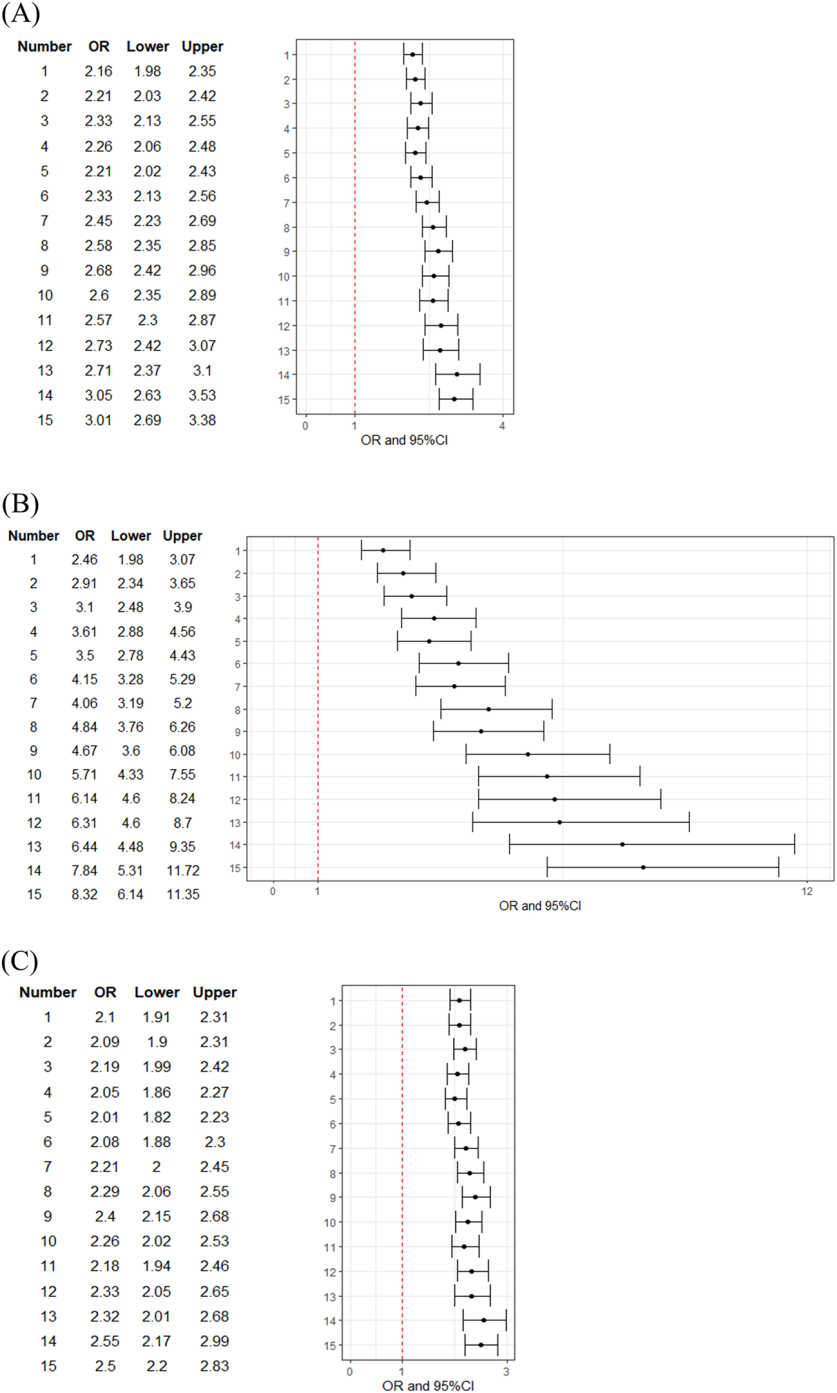
Forest plot for odds ratio on number of medications taken stratified by age of patients. OR of each number of medications taken with 0 tablets as reference for hip fracture. (**A**)All patients, 65–100 years. (**B**) Early elderly, 65–74 years. (**C**) Late elderly, 75–100 years. Number, number of medications; OR, odds ratio; Lower, lower confidence limit; Upper, upper confidence limit; CI, confidence interval.

Figure 2 shows changes in the OR of the number of medications taken with 0 as the reference to hip fracture according to each age groups categorized into the early elderly and the late elderly. Only early elderly had an increasing OR. Meanwhile, there was no increasing trend in other categories.

### Variable selection

**Figure 3:**
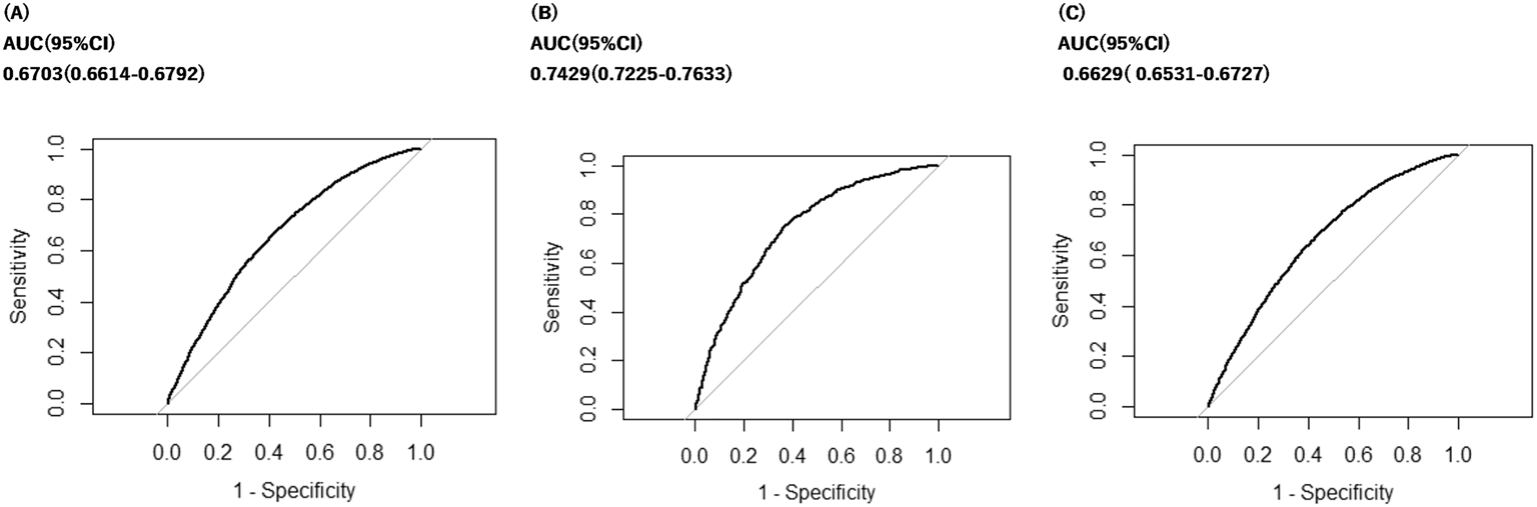
ROC curves and AUCs for the conditional LASSO. (**A**) All patients, 65–100 years. (**B**) Early elderly, 65–74 years. (**C**) Late elderly, 75–100 years. AUC, area under the curve; CI, confidence interval.

The conditional LASSO was performed using all explanatory variables for each subject population. The ROC curve analysis was done, and AUC were 0.67 (95% CI 0.66–0.68) for all elderly, 0.74 (95% CI 0.72–0.76) for the early elderly, and 0.66 (95% CI 0.65–0.67) for the late elderly (Fig. 3). The odds ratios of the selected coefficients are shown in Table 2. Of the 77 variables, 15 were selected as risk factors for all elderly, 32 for the early elderly, and 15 for the late elderly. The explanatory variable with the highest odds ratio among the selected variables was anti-Parkinson’s drugs in all the categories, followed by eye and adnexa, musculoskeletal system, and connective tissue diseases, and others.

**Table 2:** Estimated coefficients in the conditional LASSO in each age group. All patients, 65–100 years; Early elderly, 65–74 years; Late elderly, 75–100 years; OR, odds ratio. (A) Number of medications and Comorbidities. (B) Concomitant medications.

(A)			
Variable	OR		
	ALL patients	Early elderly	Late elderly
Number of medications	1.1	1.161	1.095
Certain infectious and parasitic diseases	1.064	1.15	1.065
Neoplasms	0.98	0.807	–
Diseases of the blood and blood-forming organs and certain disorders, involving the immune mechanism	–	0.937	–
Endocrine, nutritional, and metabolic diseases	0.937	1.052	0.902
Mental and behavioral disorders	1.111	1.426	1.085
Nervous system diseases	1.179	1.268	1.153
Eye and adnexa diseases	1.363	1.643	1.359
Ear and mastoid process diseases	1.131	1.127	1.191
Diseases of the circulatory system	0.768	0.822	0.738
Respiratory system diseases	–	1.233	0.993
Digestive system diseases	1.193	1.379	1.177
Skin and subcutaneous tissue diseases	1.208	1.374	1.204
Musculoskeletal system and connective tissue diseases	1.297	1.458	1.304
Genitourinary system diseases	–	1.109	–
Symptoms and signs and abnormal clinical and laboratory findings, not elsewhere classified	–	1.059	0.959
Injury, poisoning, and certain other consequences of external causes	1.184	1.124	1.142

(B)			
Variable	OR		
	ALL patients	Early elderly	Late elderly
Stomatologicals, mouth preparations, medicinal dentifrices	–	–	–
Antacids, antiflatulents, and anti-ulcers	0.764	0.77	0.765
Functional gastrointestinal disorder drugs	0.887	0.764	0.892
Antiemetics and antinauseants	–	0.457	–
Bile therapy and cholagogues	–	–	0.95
Drugs for constipation	0.615	0.47	0.627
Drugs for Intestinal disorder	0.671	0.679	0.663
Digestives system drug, including enzymes	–	1.179	–
Antidiabetic drugs	–	1.033	1.025
Vitamins	–	1.049	–
Mineral supplements	0.769	0.807	0.712
Anabolics, systemic	–	–	–
Other alimentary tract and metabolism products	–	1.184	–
Antithrombotic agents	0.934	0.942	0.957
Antifibrinolytics	–	0.585	–
Anti-anemic preparations	–	1.009	0.994
Cardiac therapy	0.848	0.689	0.927
Antihypertensives	–	–	–
Diuretics	0.861	0.981	0.879
Cerebral and peripheral vasotherapeutics	–	–	–
Antivaricosis/anti-hemorrhoidal preparations	–	–	–
Other cardiovascular products	–	0.96	–
Beta-blockers	0.969	0.852	0.931
Calcium antagonists	0.996	–	0.973
Agents acting on the renin–angiotensin system	0.958	0.912	0.963
Lipid-regulating/anti-atheroma preparations	0.881	0.739	0.879
Cardiovascular multitherapy combination products	–	–	–
Antipruritic, including topical antihistamine, anaesthetic	–	–	–
Nonsteroidal product for inflammatory skin disorders	–	–	–
Other dermatological preparations	–	–	–
Gynecological anti-infective agents	–	–	–
Sex hormones and products with similar desired effects, systemic action only	–	–	–
Urologics	–	–	–
Systemic corticosteroids	–	1.148	0.978
Thyroid therapy	–	1.009	0.975
Other types of hormones	–	1.323	–
Systemic antibacterials	0.579	0.531	0.559
Systemic agents for fungal infections	–	1.129	–
Antimycobacterials	–	–	–
Antivirals for systemic use	–	–	–
Antineoplastics	–	1.247	–
Cytostatic hormone therapy	1.198	1.221	1.266
Immunostimulating agents	–	–	–
Immunosuppressants	–	1.635	–
Anti-inflammatory and anti-rheumatic agents	0.789	0.689	0.781
Muscle relaxants	–	1.034	–
Anti-gout preparations	–	1.028	–
Other drugs for musculo-skeletal system disorders	–	–	–
Anesthetics	0.331	0.228	0.269
Analgesics	0.648	0.552	0.669
Anti-epileptics	1.001	1.454	–
Anti-Parkinson’s drugs	1.397	2.524	1.408
Psycholeptics	0.763	0.878	0.735
Psychoanaleptics, excluding anti-obesity preparations	1.006	1.329	1.061
Other Central Nervous System drugs	1.138	1.28	1.174
Antiprotozoals and anthelmintics	–	–	–
Anti-asthma and COPD products	–	–	–
Cough and cold preparations	0.855	0.703	0.853
Systemic antihistamines	–	–	–
Other respiratory system products	–	–	–

### Predictive performance

Table 3 shows the results of the conditional LASSO analysis according to all ages and each stratified age, with explanatory variables, including the number of medications alone; number of medications and comorbidities; number of medications and concomitant medications; and number of medications, comorbidities and concomitant medications. The values for the AUC, sensitivity, specificity, PPV, and NPV were calculated. Particularly, for the early elderly, the AUC was 0.61 (95% CI 0.60–0.62) when only the number of medications was used, 0.67 (95% CI 0.65–0.70) when the number of medications and each comorbidities was used, 0.71 (95% CI 0.69–0.74) when the number of medications and each concomitant medications was used, and 0.74 (95% CI 0.72–0.76) when the number of medications, each comorbidities and each concomitant medications was used.

**Table 3:** AUC and 95% CI based on the conditional LASSO for each age if the explanatory variables were varied. The conditional logistic model was used for the analysis when only the number of medications taken was used as an explanatory variable, while the other analyses were conducted using conditional LASSO. All patients, 65–100 years; Early elderly, 65–74 years; Late elderly, 75–100 years; AUC, area under the curve; CI, confidence interval; PPV, Positive Predictive Value; NPV, Negative Predictive Value. Row 1 (total 1 variable), Number of medications; Row 2 (total 17 variables), Number of medications and comorbidities (16 variables); Row 3 (total 61 variables), Number of medications and concomitant medications (60 variables); Row 4 (total 77 variables), Number of medications, comorbidities (16 variables) and concomitant medications (60 variables).

	All patients					Early elderly					Late elderly				
Explanatory variables when performing conditional LASSO or conditional logistics regression	AUC (95% CI)	Sensitivity	Specificity	PPV	NPV	AUC (95% CI)	Sensitivity	Specificity	PPV	NPV	AUC (95% CI)	Sensitivity	Specificity	PPV	NPV
Number of medications	0.54(0.53–0.54)	0.46	0.59	0.53	0.52	0.61(0.60–0.62)	0.64	0.51	0.56	0.59	0.53(0.52–0.53)	0.39	0.65	0.52	0.51
(1 variable)															
Number of medications &	0.61(0.60–0.62)	0.57	0.61	0.59	0.59	0.67(0.65–0.70)	0.69	0.6	0.63	0.66	0.6(0.59–0.61)	0.59	0.57	0.58	0.58
Presence of comorbidities															
(17 variables)															
Number of medications &	0.65(0.64–0.66)	0.58	0.62	0.61	0.6	0.71(0.69–0.74)	0.6	0.71	0.67	0.64	0.64(0.63–0.65)	0.6	0.59	0.6	0.6
Use of concomitant medications															
(61variables)															
Number of medications &	0.67(0.66–0.68)	0.66	0.61	0.63	0.64	0.74(0.72–0.76)	0.74	0.61	0.66	0.7	0.66(0.65–0.67)	0.64	0.63	0.63	0.64
Use of concomitant medications &															
Presence of comorbidities															
(77 variables)															

### Stepwise logistic analysis and external validation

Regarding variable selection, LASSO resulted in a more parsimonious selection of variables than did stepwise logistic regression analysis, enhancing interpretability. Both external validation and LASSO yielded nearly identical results. In addition, anti-Parkinson’s drugs were selected as a variable that ranked relatively high in both methods. As for the predictive performance, when considering all elderly individuals and using variables such as the number of tablets taken, various concomitant medications, and comorbidities, the AUC for the stepwise logistic regression analysis was 0.680 (95% CI, 0.671–0.6886), and for external validation, it was 0.5859 (95% CI, 0.5772–0.5945) (Appendices 5 and 6).

## Discussion

This study used a matched case–control study design for a large population-based dataset in Japan (comprising 70,000 individuals) to determine the risk factors, including polypharmacy and to establish a predictive model for hip fracture in elderly Japanese. The age distribution of hip fracture was similar to the epidemiology in Japan, with the number of women higher than that of men, peaking in the 80 s^9,12^. Polypharmacy was associated with an increased risk of hip fracture only in the early elderly and had minimal effect on hip fracture in the late elderly. The use of anti-Parkinson’s drugs showed the highest OR in the predictive model for hip fracture. Moreover, a comprehensive model including comorbidities and concomitant medications was more accurate in predicting hip fracture than a model that uses the number of medications alone. However, in older patients, the risk was more challenging to predict even with the model. Validation studies of diagnosing a disease of the claims database in Japan have been conducted in the past^25–28^ and have consistently demonstrated moderate to high sensitivity and high specificity. According to the study by Yamana et al., which has been cited in numerous papers, the validity of diagnosis in the DPC database demonstrated a sensitivity and specificity of 78.9% and 93.2%, respectively. Although variations were observed among different medical conditions, overall findings indicated favorable accuracy without significant diagnostic inaccuracies^28^.

According to Mortazavi et al., polypharmacy is defined as a condition in which more medications are administered than necessary or unnecessary medications are prescribed, although no unified definition of polypharmacy has been established in Japan or internationally^29^. The Ministry of Health, Labour and Welfare in Japan defines it as “not just a high number of medications being taken, but a condition associated with increased risk of adverse drug events, medication errors, and decreased medication adherence”^30^. Regarding numerical definitions, many studies have reached a consensus, such as the study by Masnoon et al., that the use of 5 or more drugs indicates polypharmacy^31–34^. According to the study by Lai et al. on hip fractures^35^ and Huang et al. on falls^36^, the number of medications is proportionally related to the adverse events of those medications regardless of sex or age. When multiple medications are administered, the increased risk of falls due to drug interactions, adverse drug effects, electrolyte imbalances, and reduced drug metabolism capacity can enhance the risk of hip fractures^35^. However, our results from the univariate analysis showed that, this is not applicable for all the elderly people.

The data in Appendix 4 suggested that the number of medications taken in the case and control groups was similar in all the elderly and late elderly, but there was a significant difference among the individual of the early elderly group. This outcome may have influenced the relationship between the number of medications taken and the risk of hip fractures differently in early elderly and late elderly individuals. One hypothesis for the observed difference in the number of medications taken based on age groups between the case and control groups could be that among later elderly individuals, medications containing PIMs are being appropriately managed, whereas in the early elderly, these medications might not be managed properly. The cause of polypharmacy is reportedly related to a vicious cycle called the prescribing cascade, in which taking many PIMs leads to new comorbidities and concomitant medications^37,38^. Japan has a universal health insurance system where all the citizens can access medical care. Moreover, the Japanese healthcare system differs in the extent of medical expenses between the early elderly and the late elderly. Thus, the co-payment amount is lower for the late elderly. Moreover, Torikai et al. reported that a higher percentage of the late elderly have primary care doctors than the early elderly^39^ and that the improvement rate is higher when primary care doctors are present^40^. Therefore, PIM prescriptions may be appropriately managed in Japan’s late elderly because they can frequently visit the clinic and are more likely to receive appropriate medical care. Also, since the MDV database comprises data from DPC hospitals, it may contain data from patients more severely ill than the general population. In later life, the disease may be more severe. Therefore, it may not reflect the late elderly Japanese in the general population. Therefore, we speculated that the OR did not increase with the number of medications taken in the late elderly.

Among the predictors selected from conditional LASSO, the use of anti-Parkinson’s drugs had the highest OR in all the categories. Parkinson’s disease is a neurodegenerative disease with four major motor impairments (tremor, muscle stiffness, immobility, and postural reflex disorder). As the disease progresses, difficulty walking and postural instability appear, increasing the likelihood of falls^41–43^. Therefore, many reports, including that of Nam et al., have reported Parkinson’s disease as a risk factor for hip fracture^44–47^. However, in this study, the use of anti-Parkinson’s drugs was an important risk factor in all the categories. The effect of Parkinson’s disease may have been underestimated, as we combined Parkinson's disease, Alzheimer's disease, epilepsy, and other diseases into one category as Nervous system diseases. In addition, there are several different types of anti-Parkinson’s drugs in the market, and the side effects of these drugs themselves may also cause hip fracture. These include levodopa-induced dyskinesia due to levodopa overdose, hallucinations, and delusions caused by MAO-B inhibitors and dopamine agonists, and cognitive dysfunction attributed to anticholinergic drugs^48^. Anti-Parkinson’s drugs were selected as the top variable because their side effects are directly correlated with falls. Speculatively, osteoporosis is included in the classification of “Musculoskeletal system and connective tissue diseases”; thus, it was likely chosen as a higher-level variable. However, in terms of therapeutic medications, they are categorized under “Other drugs for musculoskeletal system disorders” and were not selected as relevant factors. The reason could be the presumed preventive effect of these medications on hip fractures even in cases of falls, when treatment is administered. Furthermore, this dataset exclusively focuses on DPC hospital data and does not include information from small-scale or chronic-phase hospitals and clinics. Thus, it might not accurately represent the general patient population. This aspect serves as a limitation of this study.

Moreover, diverse prediction accuracy was observed between the early and late elderly. The influence of geriatric syndrome and frailty can be considered as the reason for our result that the prediction accuracy is the highest in patients in early elderly and the prediction accuracy becomes lower with age. Geriatric syndrome is defined as a collection of mental and physical symptoms caused by reduced mental and physical functions throughout the body^49–51^. However, frailty is the stage prior to the need for nursing care, where the decline in physiological reserve and function associated with aging leads to increased vulnerability to endogenous and exogenous stress, thereby resulting in health issues, such as functional impairment in daily life. Kojima et al. reported that prevalence increases especially in the late 70 s^52^, progressing into a vicious cycle called the frailty cycle, in which various frailty factors overlap^53^. Thus, geriatric syndrome and frailty are complex multifactorial syndromes that are caused by multiple factors, and their incidence increases with advancing age. Hence, they are not likely diagnosed as a specific disease even though they may potentially have an increased risk of comorbidities. Therefore, with older age, the predictive accuracy of hip fracture is lower, even with all of the explanatory variables applied in this study. Another reason for the lower predictive accuracy may be that the factors affecting hip fracture become more diverse as people get older.

Based on the analysis results, the predictive model with conditional LASSO for hip fracture had the lowest AUC for the model using only the number of medications taken and the highest AUC using all variables. Interpretability and predictive accuracy are a trade-off. Thus, the concept of polypharmacy is challenging to signify on the basis of the number of medications taken alone, as it increases interpretability but decreases the predictive accuracy. Polypharmacy is associated with PIM and prescribing cascades. Therefore, when hip fracture occurs, it is not only related to PIMs but also to the type of disease and concomitant medications associated with PIMs. As a result, since all the factors affect hip fracture, we speculate that using all variables compared with the number of medications taken alone could have produced a model with a higher predictive accuracy and interpretability with optimal variable selection via the use of conditional LASSO.

The current study had several limitations. First, since this data is obtained from acute care hospitals included in the administrative database (MDV), the patients are likely to be more severely ill or have more comorbidities than the general population. Additionally, since each patient was diagnosed and treated only within the same hospital, events diagnosed or medications prescribed outside the hospital related to the emergency admission may not be included in the database. For these reasons, the generalizability of data to other countries and the Japanese population as a whole might be low. Second, with regard to use of concomitant medications and presence of comorbidities, since several medications and diseases are included in one category, the specific factors affecting the patient remain unknown (such as, the endocrine, nutritional, and metabolic diseases category includes thyroid disease, diabetes, and dyslipidemia, so exposure is not uniform). Third, because this is a database study, confounding caused by unmeasured factors could not be excluded. Fourth, it is not possible to distinguish between osteoporosis-related fragility fractures and those resulting from trauma in cases of hip fractures. However, since this study specifically focuses on individuals aged ≥ 65, it is believed that osteoporosis-related fragility fractures and factors such as medication side effects and reduced muscle strength leading to falls are likely to be the primary causes, with a lower likelihood of trauma being the cause^12,54^. The characteristics of MDV data make it impossible to evaluate the following: diagnostic accuracy, detailed clinical information other than the presence of comorbidities and use of concomitant medications, and whether hip fracture is a primary or recurrent event. Finally, the existence of coding errors cannot be denied as this database was not created for academic research. Therefore, consistency with other machine learning methods is also a limitation for future studies.

However, despite these limitations, the current study had strengths that outweigh the limitations. First, we used a conditional LASSO analysis to improve the predictive accuracy and interpretability of statistical models generated by performing both variable selection and regularization on the paired data in a matched case–control study. Secondly, this study first identified the association between hip fracture and polypharmacy in a large data set of 70,000 people in Japan. We then successfully clarified the most influential factor from a large number of variables that could be the risk factor of hip fracture and the age with high prediction accuracy by conditional LASSO.

## Conclusion

Based on real-world data, no proportional relationship between the number of medications and the development of adverse drug events was observed in all age groups among elderly individuals in Japan. Moreover, in addition to the number of medications taken, many factors are associated with the hip fracture. Anti-Parkinson drugs were identified as a risk factor for hip fracture. With the population aging in Japan, the prevalence of hip fractures and their associated medical expenses will increase. Other countries with elderly populations are monitoring Japan closely to determine how it handles this issue. As a preventive strategy, physicians and pharmacists must pay special attention and prescribe appropriately to the early elderly to prevent hip fractures due to polypharmacy. Furthermore, in the late elderly, we should not only focus only on concomitant medications and comorbidities but also consider the use of a comprehensive approach based on potential factors. This survey can help in the prevention and management of hip fracture in the future, an issue that should be addressed not only in Japan but also in other countries worldwide.

## Methods

### Study design and population

This was a population-based matched case–control study. The administrative claims data provided by the Japanese MDV were used to conduct a retrospective evaluation. The data cover approximately 22% DPC hospitals, which include many acute care hospitals throughout Japan, and comprise data on electric health insurance claims, DPC claims, and laboratory test results^55^. Moreover, of the total elderly population in Japan, the data of 35.23 million elderly attending 438 DPC hospitals under contract with MDV were extracted. In our study, the case group comprised all patients diagnosed with hip fractures between April 2008 and December 2020. Considering cost feasibility, the control group did not encompass all patients with non-hip fractures but instead included randomly selected approximately one million individuals to represent Japan’s demographic dynamics. For diseases, diagnosis codes from the International Classification of Diseases, 10th Revision (ICD-10), Japanese disease codes, and dates of diagnosis were registered. For medications, Japanese treatment codes, health insurance claims codes, drug prescription dates, routes of administration, and prescribed doses were registered. All patient data were coded prior to entry.

The healthcare system in Japan categorizes the elderly population in two groups: early elderly aged < 75 years and late elderly aged > 75 years, because factors such an increase in the incidence of multiple illnesses including dementia and mortality rate among these groups are associated with different medical expense burdens. We decided to conduct additional analysis by splitting the data into two groups because we could not find any previous studies that had analyzed patients based on this categorization.

### Selection of cases and controls

For cases, the disease code in the data set was mapped to the master code of hip fracture, and the disease record identifier was the DPC claim disease name. The master code was defined based on fractures of femur (S72) from ICD-10 diagnosis codes.

As for the DPC injury/illness classification, the injury/illness that warrants greatest investment of medical resources and primary injury/illness were defined and limited to definite illnesses. Furthermore, cases were defined as patients aged ≥ 65 years who had an initial diagnosis of hip fracture between January 2015 and December 2020, and their date of diagnosis was designated as the index date. For controls, the index date was randomly set and matched 1:1 according to age and sex. Age was categorized per 5 years and was matched within that range.

### Definition of concomitant medications and comorbidities

Data on type of medications, number of medications, and presence of comorbidities were obtained and counted from the MDV database. The 9-digit health insurance claims code, which is assigned with a unique number for each medication based on its efficacy, is converted to a 5-digit code based on the Anatomical Therapeutic Chemical Classification System administered by the European Pharmaceutical Marketing Research Association^56^. Medications were limited to those that were started prior to the index date and were continually taken as of the index date or were discontinued within 7 days of the index date. The grace period was set to 7 days, and all medications taken at least once within 7 days of the index date had an effect on outcome. In addition, all medications with health insurance claims codes that were started before the index date were selected, with consideration of the 7-day gap between the start date of treatment and the next start date (i.e., the period during which prescriptions were continued). The number of medications was then calculated by summing up the amount of prescriptions for each day that all those medications were prescribed. The number of medications was set to the maximum value within 7 days of the index date as the number of medications taken during the occurrence of hip fracture.

Category of comorbidities correlated with hip fracture was restricted to diseases for which a confirmed diagnosis was established from the disease name order, and those that were assigned before the index date and for which no end date was registered by the index date. The extracted ICD-10 codes were mapped to the major categories of the Statistical Classification of Diseases, Injuries and Deaths of the Ministry of Health, Labor and Welfare^57^, which is used in statistical surveys in Japan, according to the basic classification columns of the same table, and were categorized into ICD chapters.

Based on them, data on concomitant medications, number of medications, and presence of comorbidities were collected from the MDV database. The explanatory variables were sex, age, number of tablets, 16 variables for comorbidities based on the ICD-10 codes (Appendix 1), and 60 variables for concomitant medications based on the ATC codes (Appendix 2).

### Statistical analysis

Sex, concomitant medications, and comorbidities were treated as binary variables and the number of medications taken as a continuous variable. Age was stratified as a categorical variable using a cutoff value of 75 to divide into early elderly and late elderly. The characteristics of case and control participants in terms of age, sex, number of medications and major comorbidities, and comorbidities were calculated as means, standard deviations, and percentages and were expressed as descriptive statistics.

Conditional LASSO was used to evaluate the influence of each defined variable on hip fracture and the predictive model. LASSO model is known to have many desirable properties for regression models with large covariates, such as the ability to efficiently optimize the loss of logistic regression by adding the sum of absolute values of partial regression coefficients (L1 regularization term) to the loss function of linear regression^19,20^. The logistic regression model penalized the absolute size of the regression coefficients according to the tuning parameter λ. As the penalty increases, only the strongest predictors remain in the model, and the estimates of the weaker factors are reduced to zero^58^. The conditional logistic LASSO model improves power by extending the usual logistic model to matched binary data for large high-dimensional data from individually matched case–control studies and subtracting the similarity between the pairs^59,60^. The Lasso applied to conditional logistic regression is maximizing the conditional log-likelihood function penalized by the L1 norm of the unknown coefficient vector or, equivalently, minimizing the negative objective function:

$$\underset{\beta }{\mathrm{min}}(-\mathrm{log}(L\left(\beta ,D\right)+\lambda {\Vert \beta \Vert }_{1}),$$

where $\lambda$ is a regularization parameter, and ${\| \beta \| }_{1}$=${\sum }_{j=1}^{p}|{\beta }_{j}|$ is the ${L}^{1}$- norm of coefficients^59^.

The receiver operating characteristic (ROC) curves were drawn and area under the curve (AUC) was calculated to validate the predictive performance of the model using conditional LASSO. The ROC curve is an index used to evaluate the ability of the model for predicting the risk of event occurrence in relation the actual event occurrence. The AUC is a numerical expression of this index. Then, using the ROC curve, the optimal cutoff value was obtained from the estimated Youden index (sensitivity + specificity − 1). The sensitivity, specificity, negative predictive value (NPV), and positive predictive value (PPV) were calculated at this cutoff point. The stepwise logistic regression was also performed and compared with the conditional LASSO for variable selection and AUC.

In addition, external validation of the developed predictive model was conducted using an external validation cohort consisting of patients with an index date up to December 31, 2014. All analyses were performed using R version 4.1.0. The package “clogitL1” was used for conditional LASSO analysis.

### Ethics declarations

This study was conducted in compliance with the Ethical Guidelines for Medical and Biological Research Involving Human Subjects by the Ministry of Education, Culture, Sports, Science and Technology, and the Ministry of Health, Labour and Welfare, Japan. It was approved by the Ethics Committee for Medical Research of Juntendo University School of Medicine (Research permit number E21-0264-M01).

### Patient consent for publication

According to the Ethical Guidelines for Medical and Biological Research Involving Human Subjects by the Ministry of Education, Culture, Sports, Science and Technology, and the Ministry of Health, Labour and Welfare, Japan, informed consent is not required for this study because the study uses information that has already been anonymized (anonymized processed information).

### Supplementary Information

Supplementary Information.

## Data Availability

The dataset used in our study is exclusive to Medical Data Vision, Co., Ltd. It was purchased by Juntendo University and not publicly available. Researchers who have an interest and want to use the data for research purposes may contact Dr. Nojiri (E-mail: s-nojiril@juntendo.ac.jp) to sign a data usage agreement and pay a fee to acquire the data.
