# Supplementary material for: Association between number of medications and hip fractures in Japanese elderly using conditional logistic LASSO regression
Source: Sci Rep. 2023 Oct 6;13:16831. doi: 10.1038/s41598-023-43876-3 (PMC10558461; doi:10.1038/s41598-023-43876-3)
Supplement: Supplementary file 1 — Supplementary Information. [file 41598_2023_43876_MOESM1_ESM.pdf]

Indexed according to the ICD-10 codes (chapter)	Category of complications
A00-B99	Certain infectious and parasitic diseases
C00-D48	Neoplasms
D50-D89	Diseases of the blood and blood-forming organs and certain disorders involving the immune mechanism
E00-E90	Endocrine, nutritional, and metabolic diseases
F00-F99	Mental and behavioral disorders
G00-G99	Nervous system diseases
H00-H59	Eye and adnexa diseases
H60-H95	Ear and mastoid process diseases
I00-I99	Circulatory system diseases
J00-J99	Respiratory system diseases
K00-K93	Digestive system diseases
L00-L99	Skin and subcutaneous tissue diseases
M00-M99	Musculoskeletal system and connective tissue diseases
N00-N99	Genitourinary system diseases
R00-R99	Symptoms and signs and abnormal clinical and laboratory findings, not elsewhere classified
S00-T98	Injury, poisoning, and certain other consequences of external causes

Appendix 1.

Complication classification based on the Statistical Classification of Diseases, Injuries and Deaths of the Ministry of Health, Labour and Welfare and ICD-10 codes

ICD-10, International Classification of Diseases, 10th Revision

Indexed according to ATC codes	Category of concomitant medications
A01	Stomatologicals, mouth preparations, medicinal dentifrices etc
A02	Antacids, antiflatulents, and anti-ulcers
A03	Functional gastrointestinal disorder drugs
A04	Antiemetics and antinauseants
A05	Bile therapy and cholagogues
A06	Drugs for constipation
A07	Intestinal disorder products
A09	Digestives, including enzymes
A10	Drugs used in diabetes
A11	Vitamins
A12	Mineral supplements
A14	Anabolics, systemic
A16	Other alimentary tract and metabolism products
B01	Antithrombotic agents
B02	Antifibrinolytics
B03	Anti-anemic preparations
C01	Cardiac therapy
C02	Antihypertensives
C03	Diuretics
C04	Cerebral and peripheral vasotherapeutics
C05	Antivaricosis/anti-hemorrhoidal preparations
C06	Other cardiovascular products
C07	Beta-blocking agents
C08	Calcium antagonists
C09	Agents acting on the renin-angiotensin system
C10	Lipid-regulating/anti-atheroma preparations
C11	Cardiovascular multitherapy combination products
D04	Anti-pruritics, including topical antihistamines, anaesthetics, etc
D05	Nonsteroidal products for inflammatory skin disorders
D11	Other dermatological preparations
G01	Gynecological anti-infectives
G03	Sex hormones and products with similar desired effects, systemic action only
G04	Urologics
H02	Systemic corticosteroids
H03	Thyroid therapy

H04	Other types of hormones
J01	Systemic antibacterials
J02	Systemic agents for fungal infections
J04	Antimycobacterials
J05	Antivirals for systemic use
L01	Antineoplastics
L02	Cytostatic hormone therapy
L03	Immunostimulating agents
L04	Immunosuppressants
M01	Anti-inflammatory and anti-rheumatic products
M03	Muscle relaxants
M04	Anti-gout preparations
M05	Other drugs for musculo-skeletal system disorders
N01	Anesthetics
N02	Analgesics
N03	Anti-epileptics
N04	Anti-Parkinson's drugs
N05	Psycholeptics
N06	Psychoanaleptics excluding anti-obesity preparations
N07	Other CNS drugs
P01	Antiprotozoals and anthelmintics
R03	Anti-asthma and copd products
R05	Cough and cold preparations
R06	Systemic antihistamines
R07	Other respiratory system products

Appendix 2.

Concomitant medication classification based on health insurance claims code and ATC codes.

ATC, Anatomical Therapeutic Chemical Classification System

(A) early elderly

	Case group (n = 5516)	Control group (n = 5516)
	N	N
Sex		
Male	1984 (36.0%)	1984 (36.0%)
Female	3532 (64.0%)	3532 (64.0%)
Comorbidities		
Circulatory system diseases	4176 (75.7%)	3869 (70.1%)
Digestive system diseases	4041 (73.3%)	3207 (58.1%)
Endocrine, nutritional, and metabolic diseases	3704 (67.2%)	3087 (56.0%)
Neoplasms	3243 (58.8%)	3069 (55.6%)
Respiratory system diseases	3095 (56.1%)	2274 (41.2%)
Certain infectious and parasitic diseases	2727 (49.4%)	2096 (38.0%)
Symptoms and signs and abnormal clinical and laboratory findings, not elsewhere classified	2710 (49.1%)	2048 (37.1%)
Genitourinary system diseases	2527 (45.8%)	1875 (34.0%)
Nervous system diseases	2398 (43.5%)	1399 (25.4%)
Diseases of the blood and blood-forming organs and certain disorders involving the immune mechanism	2374 (43.0%)	2025 (36.7%)
Injury, poisoning, and certain other consequences of external causes	2011 (36.5%)	1444 (26.2%)
Skin and subcutaneous tissue diseases	1897 (34.4%)	1080 (19.6%)
Eye and adnexa diseases	1580 (28.6%)	777 (14.1%)
Musculoskeletal system and connective tissue diseases	1552 (28.1%)	877 (15.9%)
Mental and behavioral disorders	1254 (22.7%)	618 (11.2%)
Ear and mastoid process diseases	558 (10.1%)	334 (6.1%)
Comcomitant medications		
Antacids, antiflatulents, and anti-ulcers	3207 (58.1%)	3169 (57.5%)
Antithrombotic agents	1602 (29.0%)	1331 (24.1%)
Agents acting on the renin-angiotensin system	1498 (27.2%)	1176 (21.3%)
Calcium antagonists	1485 (26.9%)	1188 (21.5%)

Lipid-regulating/anti-atheroma preparations	1371 (24.9%)	1189 (21.6%)
Psycholeptics	1305 (23.7%)	1092 (19.8%)
Drugs used in diabetes	1159 (21.0%)	760 (13.8%)
Vitamins	1022 (18.5%)	633 (11.5%)
Anti-inflammatory and anti-rheumatic products	970 (17.6%)	1182 (21.4%)
Diuretics	873 (15.8%)	600 (10.9%)
Drugs for constipation	846 (15.3%)	1180 (21.4%)
Anti-epileptics	808 (14.6%)	403 (7.3%)
Analgesics	716 (13.0%)	927 (16.8%)
Beta-blocking agents	698 (12.7%)	591 (10.7%)
Systemic corticosteroids	632 (11.5%)	441 (8.0%)
Anti-gout preparations	538 (9.8%)	329 (6.0%)
Intestinal disorder products	469 (8.5%)	514 (9.3%)
Systemic antibacterials	423 (7.7%)	640 (11.6%)
Other central nervous system drugs	422 (7.7%)	191 (3.5%)
Cough and cold preparations	392 (7.1%)	409 (7.4%)
Number of medications		
0	130 (2.4%)	456 (8.3%)
1	671 (12.2%)	956 (17.3%)
2	631 (11.4%)	760 (13.8%)
3	544 (9.9%)	615 (11.1%)
4	527 (9.6%)	512 (9.3%)
5	477 (8.6%)	478 (8.7%)
6	431 (7.8%)	364 (6.6%)
7	374 (6.8%)	323 (5.9%)
8	341 (6.2%)	247 (4.5%)
9	293 (5.3%)	220 (4.0%)
10	257 (4.7%)	158 (2.9%)
11	224 (4.1%)	128 (2.3%)
12	169 (3.1%)	94 (1.7%)
13	112 (2.0%)	61 (1.1%)
14	105 (1.9%)	47 (0.9%)
≥ 15	230 (4.2%)	97 (1.8%)
Number of medications		
Mean (SD)	5.9 (4.4)	4.4 (3.7)
Number of comorbidities		

Mean (SD)	7.2 (3.6)	5.4(3.0)

(B)late elderly

	Case group (n = 29201)	Control group (n = 29201)
	N	N
Sex		
Male	7936 (27.2%)	7936 (27.1%)
Female	21265 (72.8%)	21254 (72.8%)
Comorbidities		
Circulatory system diseases	23132 (79.2%)	23578 (80.7%)
Digestive system diseases	21574 (73.9%)	19688 (67.4%)
Endocrine, nutritional, and metabolic diseases	19050 (65.2%)	18557 (63.5%)
Respiratory system diseases	17085 (58.5%)	16128 (55.2%)
Neoplasms	16203 (55.5%)	15325 (52.5%)
Symptoms and signs and abnormal clinical and laboratory findings, not elsewhere classified	14930 (51.1%)	14119 (48.4%)
Genitourinary system diseases	13958 (47.8%)	12857 (44.0%)
Certain infectious and parasitic diseases	13777 (47.2%)	12183 (41.7%)
Nervous system diseases	13450 (46.1%)	11163 (38.2%)
Diseases of the blood and blood-forming organs and certain disorders involving the immune mechanism	12223 (41.9%)	11491 (39.4%)
Injury, poisoning, and certain other consequences of external causes	12044 (41.2%)	10040 (34.4%)
Skin and subcutaneous tissue diseases	10143 (34.7%)	7606 (26.0%)
Musculoskeletal system and connective tissue diseases	8661 (29.7%)	6160 (21.1%)
Eye and adnexa diseases	7934 (27.2%)	5367 (18.4%)
Mental and behavioral disorders	7683 (26.3%)	6437 (22.0%)
Ear and mastoid process diseases	3764 (12.9%)	2484 (8.5%)
Comcomitant medications		

Antacids, antiflatulents, and anti-ulcers	17068 (58.5%)	18157 (62.2%)
Antithrombotic agents	10024 (34.3%)	9757 (33.4%)
Calcium antagonists	9318 (31.9%)	8915 (30.5%)
Agents acting on the renin-angiotensin system	8161 (27.9%)	7820 (26.8%)
Psycholeptics	7163 (24.5%)	8181 (28.0%)
Diuretics	6706 (23.0%)	6878 (23.6%)
Lipid-regulating/anti-atheroma preparations	6355 (21.8%)	6217 (21.3%)
Vitamins	5488 (18.8%)	4663 (16.0%)
Drugs for constipation	4878 (16.7%)	6860 (23.5%)
Drugs used in diabetes	4163 (14.3%)	3297 (11.3%)
Beta-blocking agents	4119 (14.1%)	4038 (13.8%)
Other central nervous system drugs	4021 (13.8%)	2923 (10.0%)
Analgesics	4004 (13.7%)	5523 (18.9%)
Anti-inflammatory and anti-rheumatic products	3990 (13.7%)	4732 (16.2%)
Urologics	3016 (10.3%)	2485 (8.5%)
Anti-gout preparations	2800 (9.6%)	2474 (8.5%)
Cardiac therapy	2741 (9.4%)	2803 (9.6%)
Anti-epileptics	2464 (8.4%)	2136 (7.3%)
Intestinal disorder products	2342 (8.0%)	3334 (11.4%)
Cough and cold preparations	2330 (8.0%)	2635 (9.0%)
Number of medications		
0	805 (2.8%)	1693 (5.8%)
1	3739 (12.8%)	3743 (12.8%)
2	3245 (11.1%)	3262 (11.2%)
3	2798 (9.6%)	2681 (9.2%)
4	2605 (8.9%)	2671 (9.1%)
5	2412 (8.3%)	2520 (8.6%)
6	2349 (8.0%)	2373 (8.1%)
7	2254 (7.7%)	2143 (7.3%)
8	1997 (6.8%)	1832 (6.3%)
9	1728 (5.9%)	1514 (5.2%)
10	1402 (4.8%)	1307 (4.5%)
11	1036 (3.5%)	998 (3.4%)
12	813 (2.8%)	734 (2.5%)
13	586 (2.0%)	531 (1.8%)
14	453 (1.6%)	374 (1.3%)

≥ 15	979 (3.4%)	825 (2.8%)
Number of medications		
Mean (SD)	5.8 (4.1)	5.4 (4.1)
Number of comorbidities		
Mean (SD)	7.4 (3.6)	6.6 (3.3)

Appendix 3. Characteristics of hip fracture patients and controls （in the early elderly and late elderly）

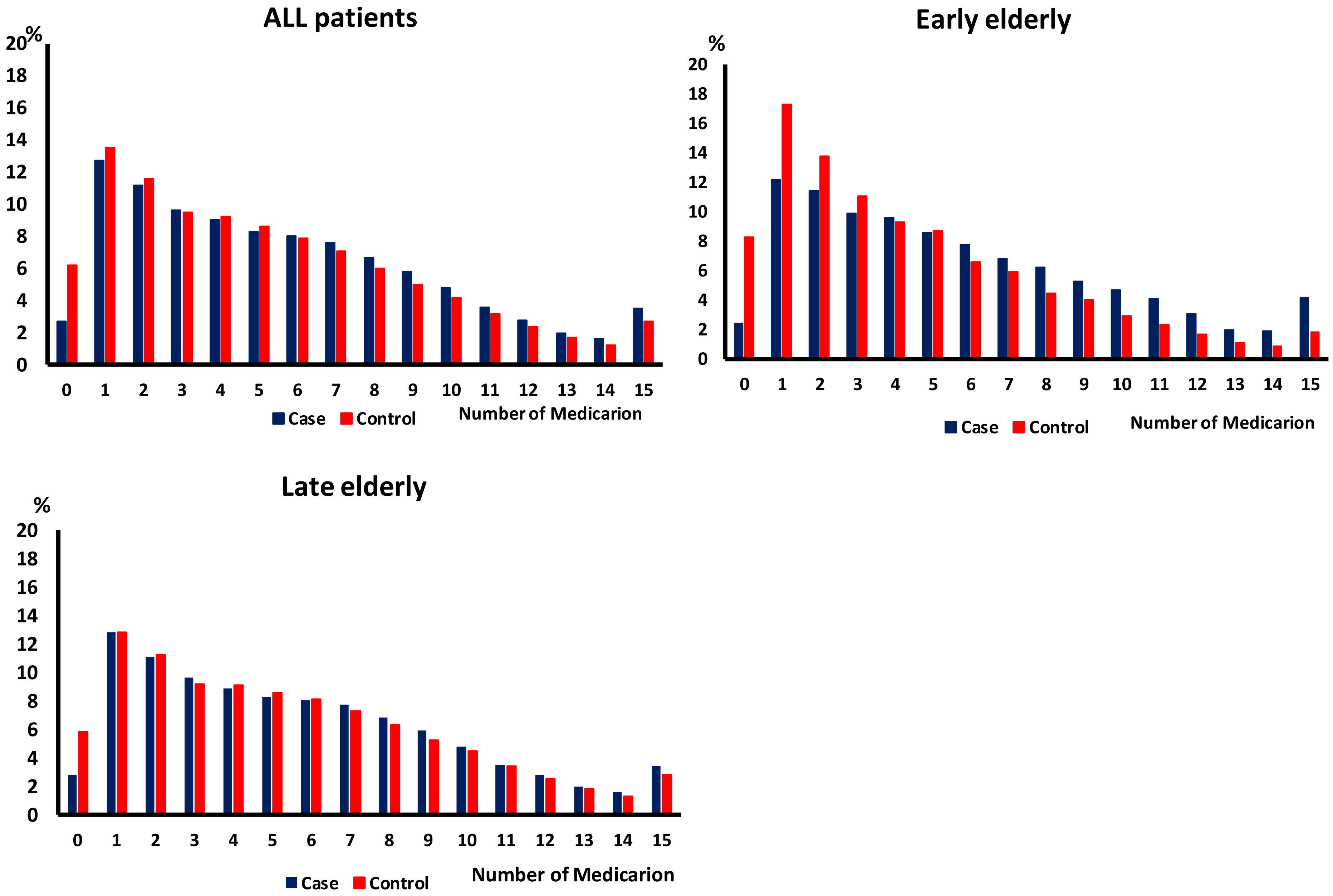

Appendix 4. Percentage distribution of the number of drugs according to each age group in the case and control groups.

ALL patients, 65-100 years; Early elderly, 65-74 years; Late elderly, 75-100 years.

Variable	OR
	ALL patients
**Number of medications**	**1.136**
**Comorbidities**	
**Certain infectious and parasitic diseases**	**1.111**
**Neoplasms**	**0.941**
**Diseases of the blood and blood-forming organs and certain disorders, involving the immune mechanism**	**-**
**Endocrine, nutritional, and metabolic diseases**	**0.9**
**Mental and behavioral disorders**	**1.138**
**Nervous system diseases**	**1.163**
**Eye and adnexa diseases**	**1.378**
**Ear and mastoid process diseases**	**1.232**
**Diseases of the circulatory system**	**0.729**
**Respiratory system diseases**	**-**
**Digestive system diseases**	**1.261**
**Skin and subcutaneous tissue diseases**	**1.238**
**Musculoskeletal system and connective tissue diseases**	**1.322**
**Genitourinary system diseases**	**0.972**
**Symptoms and signs and abnormal clinical and laboratory findings, not elsewhere classified**	**0.963**
**Injury, poisoning, and certain other consequences of external causes**	**1.166**
**Concomitant medications**	
**Stomatologicals, mouth preparations, medicinal dentifrices**	**0.676**
**Antacids, antiflatulents, and anti-ulcers**	**0.77**
**Functional gastrointestinal disorder drugs**	**0.757**
**Antiemetics and antinauseants**	**0.355**
**Bile therapy and cholagogues**	**0.868**
**Drugs for constipation**	**0.578**
**Drugs for Intestinal disorder**	**0.621**
**Digestives system drug, including enzymes**	**1.205**
**Antidiabetic drugs**	**-**
**Vitamins**	**-**
**Mineral supplements**	**0.653**
**Anabolics, systemic**	**0.573**
**Other alimentary tract and metabolism products**	**-**
**Antithrombotic agents**	**0.89**

**Antifibrinolytics**	**0.786**
**Anti-anemic preparations**	**0.936**
**Cardiac therapy**	**0.812**
**Antihypertensives**	**-**
**Diuretics**	**0.817**
**Cerebral and peripheral vasotherapeutics**	**1.111**
**Antivaricosis/anti-hemorrhoidal preparations**	**0.517**
**Other cardiovascular products**	**0.547**
**Beta-blockers**	**0.901**
**Calcium antagonists**	**0.94**
**Agents acting on the renin–angiotensin system**	**0.911**
**Lipid-regulating/anti-atheroma preparations**	**0.799**
**Cardiovascular multitherapy combination products**	**-**
**Antipruritic, including topical antihistamine, anaesthetic**	**1.717**
**Nonsteroidal product for inflammatory skin disorders**	**-**
**Other dermatological preparations**	**-**
**Gynecological anti-infective agents**	**0.191**
**Sex hormones and products with similar desired effects, systemic action only**	**-**
**Urologics**	**-**
**Systemic corticosteroids**	**0.936**
**Thyroid therapy**	**0.847**
**Other types of hormones**	**1.384**
**Systemic antibacterials**	**0.541**
**Systemic agents for fungal infections**	**0.791**
**Antimycobacterials**	**1.75**
**Antivirals for systemic use**	**0.819**
**Antineoplastics**	**1.215**
**Cytostatic hormone therapy**	**1.456**
**Immunostimulating agents**	**-**
**Immunosuppressants**	**1.407**
**Anti-inflammatory and anti-rheumatic agents**	**0.719**
**Muscle relaxants**	**-**
**Anti-gout preparations**	**-**
**Other drugs for musculo-skeletal system disorders**	**-**
**Anesthetics**	**0.159**
**Analgesics**	**0.623**
**Anti-epileptics**	**-**
**Anti-Parkinson’s drugs**	**1.604**
**Psycholeptics**	**0.707**

**Psychoanaleptics, excluding anti-obesity preparations**	**1.115**
**Other Central Nervous System drugs**	**1.173**
**Antiprotozoals and anthelmintics**	**-**
**Anti-asthma and COPD products**	**-**
**Cough and cold preparations**	**0.781**
**Systemic antihistamines**	**0.895**
**Other respiratory system products**	**0.456**

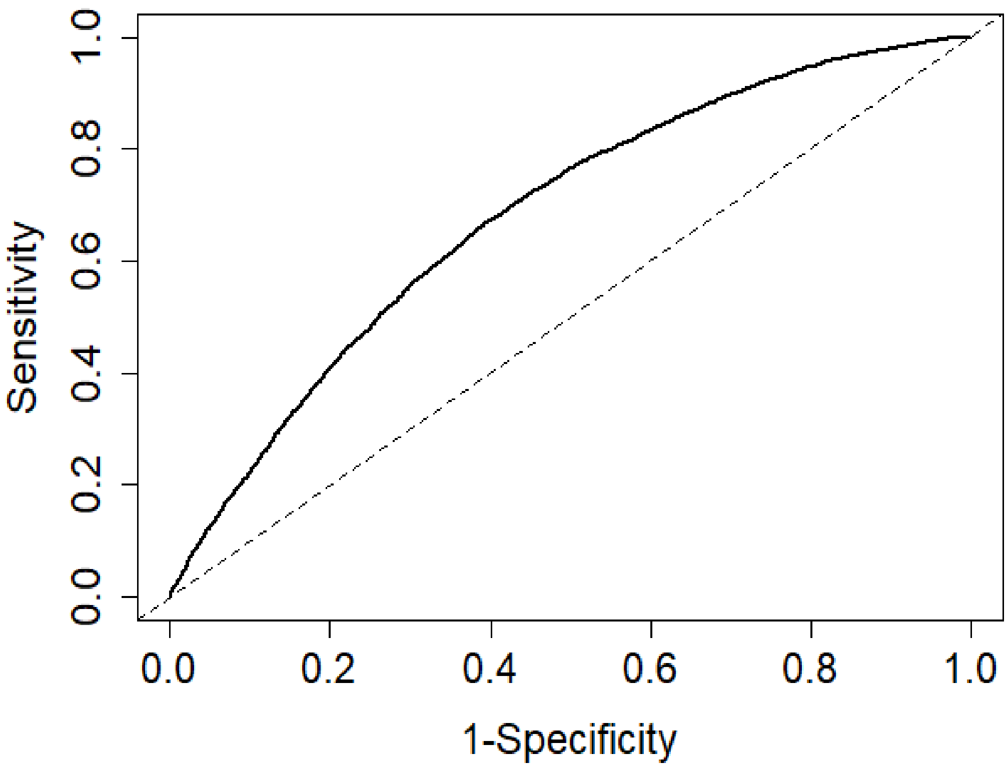

AUC(95%CI)
0.680(0.671-0.6886)

Appendix 5. Stepwise Logistic Analysis
(Variable selection and Predictive performance)

Variable	OR
	ALL patients
**Number of medications**	**1.096**
**Comorbidities**	
**Certain infectious and parasitic diseases**	**1.072**
**Neoplasms**	**0.987**
**Diseases of the blood and blood-forming organs and certain disorders, involving the immune mechanism**	**-**
**Endocrine, nutritional, and metabolic diseases**	**0.922**
**Mental and behavioral disorders**	**1.086**
**Nervous system diseases**	**1.167**
**Eye and adnexa diseases**	**1.375**
**Ear and mastoid process diseases**	**1.098**
**Diseases of the circulatory system**	**0.769**
**Respiratory system diseases**	**-**
**Digestive system diseases**	**1.207**
**Skin and subcutaneous tissue diseases**	**1.227**
**Musculoskeletal system and connective tissue diseases**	**1.296**
**Genitourinary system diseases**	**-**
**Symptoms and signs and abnormal clinical and laboratory findings, not elsewhere classified**	**-**
**Injury, poisoning, and certain other consequences of external causes**	**1.15**
**Concomitant medications**	
**Stomatologicals, mouth preparations, medicinal dentifrices**	**-**
**Antacids, antiflatulents, and anti-ulcers**	**0.787**
**Functional gastrointestinal disorder drugs**	**0.873**
**Antiemetics and antinauseants**	**-**
**Bile therapy and cholagogues**	**-**
**Drugs for constipation**	**0.615**
**Drugs for Intestinal disorder**	**0.669**
**Digestives system drug, including enzymes**	**-**
**Antidiabetic drugs**	**1.026**
**Vitamins**	**-**
**Mineral supplements**	**0.779**
**Anabolics, systemic**	**-**
**Other alimentary tract and metabolism products**	**-**
**Antithrombotic agents**	**0.955**

**Antifibrinolytics**	**-**
**Anti-anemic preparations**	**-**
**Cardiac therapy**	**0.894**
**Antihypertensives**	**-**
**Diuretics**	**0.857**
**Cerebral and peripheral vasotherapeutics**	**-**
**Antivaricosis/anti-hemorrhoidal preparations**	**-**
**Other cardiovascular products**	**-**
**Beta-blockers**	**0.983**
**Calcium antagonists**	**0.992**
**Agents acting on the renin–angiotensin system**	**0.967**
**Lipid-regulating/anti-atheroma preparations**	**0.874**
**Cardiovascular multitherapy combination products**	**-**
**Antipruritic, including topical antihistamine, anaesthetic**	**-**
**Nonsteroidal product for inflammatory skin disorders**	**-**
**Other dermatological preparations**	**-**
**Gynecological anti-infective agents**	**-**
**Sex hormones and products with similar desired effects, systemic action only**	**-**
**Urologics**	**-**
**Systemic corticosteroids**	**-**
**Thyroid therapy**	**-**
**Other types of hormones**	**-**
**Systemic antibacterials**	**0.579**
**Systemic agents for fungal infections**	**-**
**Antimycobacterials**	**-**
**Antivirals for systemic use**	**-**
**Antineoplastics**	**-**
**Cytostatic hormone therapy**	**1.157**
**Immunostimulating agents**	**-**
**Immunosuppressants**	**-**
**Anti-inflammatory and anti-rheumatic agents**	**0.803**
**Muscle relaxants**	**-**
**Anti-gout preparations**	**-**
**Other drugs for musculo-skeletal system disorders**	**-**
**Anesthetics**	**0.346**
**Analgesics**	**0.671**
**Anti-epileptics**	**1.012**
**Anti-Parkinson’s drugs**	**1.427**
**Psycholeptics**	**0.783**

**Psychoanaleptics, excluding anti-obesity preparations**	**1.038**
**Other Central Nervous System drugs**	**1.135**
**Antiprotozoals and anthelmintics**	**-**
**Anti-asthma and COPD products**	**-**
**Cough and cold preparations**	**0.89**
**Systemic antihistamines**	**-**
**Other respiratory system products**	**-**

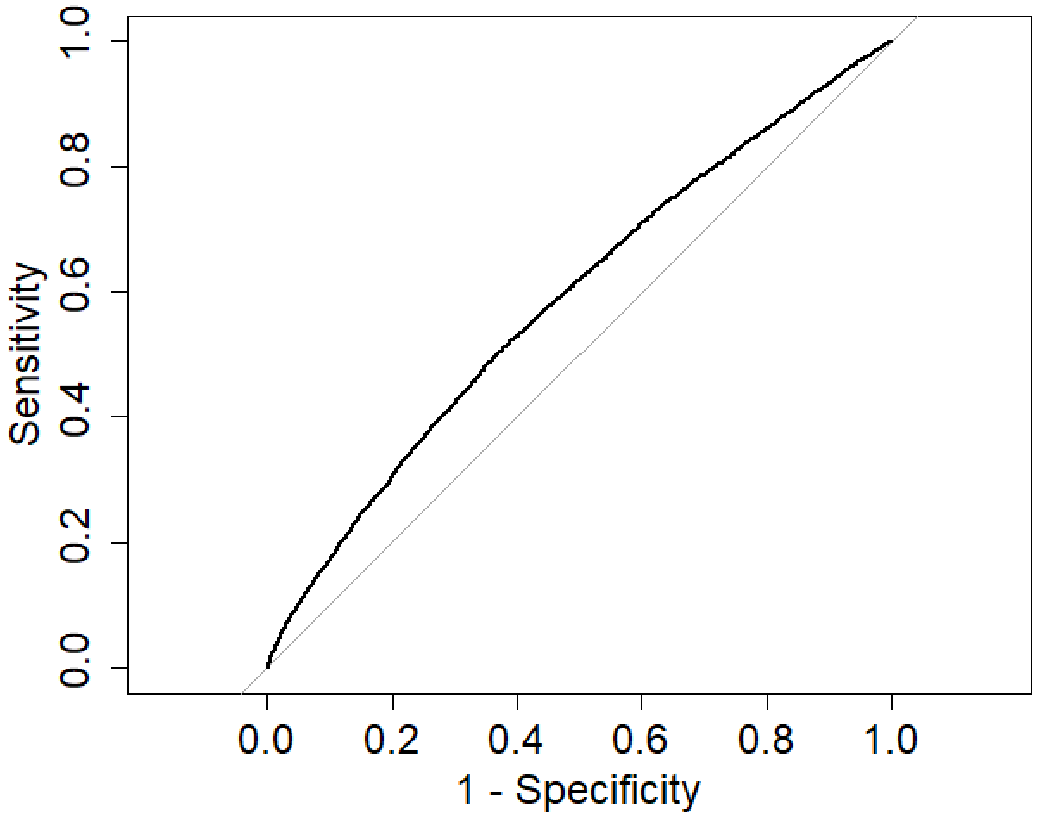

AUC(95%CI)
0.5859(0.5772-0.5945)

Appendix 6. External Validation
(Variable selection and Predictive performance)
